# *Chlamydia pneumoniae* and Oxidative Stress in Cardiovascular Disease: State of the Art and Prevention Strategies

**DOI:** 10.3390/ijms16010724

**Published:** 2014-12-30

**Authors:** Marisa Di Pietro, Simone Filardo, Fiorenzo De Santis, Paola Mastromarino, Rosa Sessa

**Affiliations:** Department of Public Health and Infectious Diseases, “Sapienza” University, Rome 00185, Italy; E-Mails: marisa.dipietro@uniroma1.it (M.D.P.); simone.filardo@uniroma1.it (S.F.); fiorenzo.desantis@uniroma1.it (F.D.S.); paola.mastromarino@uniroma1.it (P.M.)

**Keywords:** *C. pneumoniae*, cardiovascular disease, oxidative stress, prevention strategies

## Abstract

*Chlamydia pneumoniae*, a pathogenic bacteria responsible for respiratory tract infections, is known as the most implicated infectious agent in atherosclerotic cardiovascular diseases (CVDs). Accumulating evidence suggests that *C. pneumoniae-*induced oxidative stress may play a critical role in the pathogenesis of CVDs. Indeed, the overproduction of reactive oxygen species (ROS) within macrophages, endothelial cells, platelets and vascular smooth muscle cells (VSMCs) after *C. pneumoniae* exposure, has been shown to cause low density lipoprotein oxidation, foam cell formation, endothelial dysfunction, platelet adhesion and aggregation, and VSMC proliferation and migration, all responsible for the typical pathological changes of atherosclerotic plaque. The aim of this review is to improve our insight into *C. pneumoniae*-induced oxidative stress in order to suggest potential strategies for CVD prevention. Several antioxidants, acting on multi-enzymatic targets related to ROS production induced by *C. pneumoniae*, have been discussed. A future strategy for the prevention of *C. pneumoniae*-associated CVDs will be to target chlamydial HSP60, involved in oxidative stress.

## 1. Introduction

Cardiovascular disease (CVD) is a major health problem in developed countries with over 17 million deaths per year [1] and the main pathological process underlying this disease is the atherosclerosis.

Atherosclerosis typically begins with endothelial injury followed by low-density lipoprotein (LDL) oxidation and accumulation within vascular cells, triggering the pro-inflammatory cascade [interleukin (IL)-1, IL-6 and tumor necrosis factor (TNF)-α] and the subsequent proliferation of smooth muscle cells. Through this complex process, a sequence of events, including foam cell formation followed by fibrous cap and thrombus formation in the advanced plaque occurs leading to cardiovascular diseases, such as coronary heart diseases (angina, myocardial infarction), stroke, and peripheral vascular diseases [2].

Current opinion is that the most implicated infectious agent in the pathogenesis of CVDs is *Chlamydia pneumoniae*, known as the etiologic agent of respiratory tract infections [3,4]. *C. pneumoniae* is an intracellular obligate pathogen with a unique developmental cycle, characterized by two alternating functionally and morphologically distinct forms: The elementary body, the metabolically inert and infectious form, and the reticulate body, the intracellular replicative form. In the last years, the attention has been drawn to a third non-replicating and non-infectious form, called persistent form, described as involved in the pathogenesis of chronic inflammatory diseases such as atherosclerosis. Indeed, chlamydial persistent form may endure for a long time inside host cells, since it is able to evade the host immune response leading to a chronic inflammatory state in the vascular wall [5,6,7].

The relationship between *C. pneumoniae* and CVDs has been first suggested in 1988 by Saikku* et al.* [8]. Since then, increasing evidence has supported the involvement of *C. pneumoniae* in the pathogenesis of CVDs. The association has been well documented by seroepidemiological studies [4,9,10], direct detection of microorganism within atherosclerotic plaque [9,11,12,13] and* in vivo* studies showing the ability of *C. pneumoniae* to disseminate from lungs to extrapulmonary sites, such as the vascular wall, by peripheral blood mononuclear cells [14,15,16,17]. Stronger evidence on the matter came, mainly, from the isolation of viable *C. pneumoniae* from the atheroma [18,19,20] and by experimental studies showing an atherosclerotic lesion exacerbation following the *C. pneumoniae* inoculation of hyperlipidemic animal models [21,22,23,24].

Despite extensive evidence on the association between *C. pneumoniae* and CVDs, there are still issues to be addressed, such as the lack of well-standardized and adequately validated diagnostic tests for detecting *C. pneumoniae*. This problem is made more difficult for the presence of *C. pneumoniae* persistent forms, which are not consistently detectable by currently used methods. Surely, in the future, a deep and complete analysis of the genome of *C. pneumoniae* from CVD patients will more likely increase our understanding of the pathogenetic mechanisms underlying the development of atherosclerosis.

In fact, the mechanisms by which *C. pneumoniae* may influence the atherogenesis have yet to be fully clarified. Initially, it has been hypothesized that *C. pneumoniae* may contribute to atherosclerosis through inflammation, as evidenced by an increased production of inflammatory cytokines (IL1-β, IL-6, IL-8 and TNF-α) and chemokines found in vascular cells involved in the atherosclerotic process [25,26,27,28,29,30]. This hypothesis has been further confirmed by increased levels of inflammatory markers, observed in patients with CVDs and *C. pneumoniae* infection [31,32,33].

More recently, it is believed that *C. pneumoniae* may contribute to atherosclerosis through oxidative stress, as suggested by an increased production of reactive oxygen species (ROS) following *C. pneumoniae* interaction with vascular cells [34].

The oxidative stress, characterized by ROS overproduction, was demonstrated for the first time in *C. pneumoniae* infected macrophages, leading to LDL oxidation and foam cell formation. The increased production of ROS, induced by *C. pneumoniae*, has also been demonstrated in platelets, endothelial cells and vascular smooth muscle cells (VSMCs) causing both LDL oxidation and further pro-atherogenic effects, such as endothelial dysfunction, VSMC proliferation and migration, and platelet adhesion and aggregation [34].

In addition, there is also the evidence that *C. pneumoniae* and oxidized LDL (oxLDL) induce necrosis in macrophages as well as in endothelial cells, thus contributing to vascular inflammation and progression of atherosclerotic lesion [35,36].

The growing body of evidence linking *C. pneumoniae* to atherosclerosis through the elicitation of oxidative stress in the vascular wall raises interesting questions concerning the possibilities of CVD prevention by limiting ROS production and/or accelerating their inactivation. Therefore the aim of this review is to improve our insight into *C. pneumoniae*-induced oxidative stress in order to suggest potential strategies for the prevention of CVDs.

## 2. *Chlamydia pneumoniae*-Induced Oxidative Stress

In the vascular wall, ROS are produced by several enzyme systems including NADPH oxidase (NOX), xanthine oxidase (XO), uncoupled endothelial nitric oxide synthase (eNOS) and the mitochondrial electron transport chain. On the other hand, the vasculature is protected by antioxidant enzyme systems, including superoxide dismutases (SOD), catalase, glutathione peroxidases (GPx) and paraoxonases, which detoxify ROS. ROS, including free oxygen radicals, oxygen ions and peroxides, under physiological conditions, act as signaling molecules and play an important role in the regulation of vascular tone, cell growth and proliferation, apoptosis, and inflammatory responses. When the release of ROS is not limited by antioxidant defense systems, oxidative stress promotes atherogenesis through several mechanisms, including lipoprotein and phospholipid oxidation [37,38].

Generally speaking, macrophages, endothelial cells, VSMCs and platelets involved in atherosclerotic process may all be a source of ROS through different enzymatic pathways [38]. In macrophages, endothelial cells and VSMCs, ROS are produced by NOX activity and mitochondrial respiratory electron transport chain. In endothelial cells, under pathological conditions associated to oxidative stress, ROS are also produced by uncoupled eNOS activity, potentiating the preexisting oxidative stress. Lastly, in platelets, ROS production seems to depend on cyclooxygenase (COX), lipoxygenase (LOX), NOX and nitric oxide synthase (NOS) activities [39].

**Figure 1:**
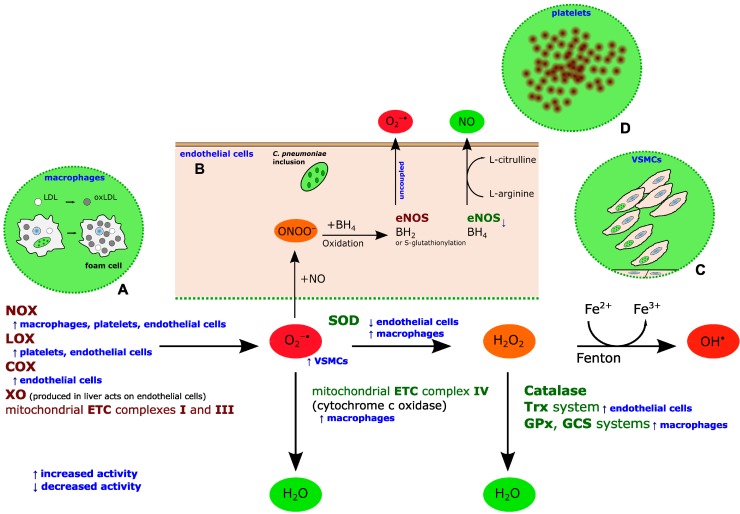
ROS generation and inactivation in the vascular wall and the contribution of *C. pneumoniae*. Generally, superoxide anion (O**_2_^−·^**) can be produced by NADPH oxidase (NOX), lipoxygenases (LOX), cycloxygenases (COX), xanthine oxidase (XO) and mitochondrial electron transport chain (ETC) complexes I and III during the oxidative respiration. O**_2_^−·^** can be disproportionated to hydrogen peroxide (H_2_O_2_) by superoxide dismutase (SOD). H_2_O_2_ can spontaneously convert to hydroxyl radical (OH**^·^**) via the Fenton reaction. Alternatively, H_2_O_2_ can be detoxified to water via catalase, glutathione peroxidase (GPx) and γ glutamylcysteine synthase (GCS) or thioredoxin (Trx) systems. O**_2_^−·^** can also be directly detoxificated into water by cytochrome c oxidase (mitochondrial ETC complex IV). Under pathological conditions associated to oxidative stress, superoxide anion (O**_2_^−·^**) may react with nitric oxide (NO), produced by endothelial nitric oxide synthase (eNOS), yielding peroxynitrite (ONOO**^−^**), which oxidizes the tetrahydrobiopterin (BH_4_) cofactor. Therefore, the BH_4_ deficiency leads to eNOS uncoupling with consequent release of O**_2_^−·^** instead of NO. In addition, under oxidative stress eNOS uncoupling may occur following to eNOS S glutathionylation. *C. pneumoniae* promotes oxidative stress through different enzymatic pathways: in macrophages, it enhances both NOX activity and activities of detoxification systems such as mitochondrial ETC complex IV (cytochrome c oxidase), SOD, GPx and GCS; in platelets, it enhances NOX and LOX activities; in VSMCs, ROS production is elicited in a NOX-independent way; in endothelial cells, it enhances NOX, LOX and COX activities, while reducing SOD, Trx and eNOS activities. Blue arrows refer to *C. pneumoniae* effect on the related enzyme activity. Overall, *C. pneumoniae-*induced oxidative stress contributes to atherogenesis, leading to: (**A**) intracellular survival of *C. pneumoniae* inside macrophages, LDL oxidation and foam cell formation; (**B**) endothelial dysfunction, characterized by increased production of anion superoxide and reduced NO bioavailability; (**C**) VSMC proliferation and migration; and (**D**) platelet activation and aggregation.

An enhanced ROS accumulation in all the cells involved in the atherosclerotic process has been demonstrated following the exposure to *C. pneumoniae* [34] (Figure 1).

In macrophages, *C. pneumoniae* has been shown to elicit superoxide anion production via NOX pathway and, at the same time, increase the antioxidant activity of cytochrome c oxidase, which may paradoxically attenuate the ROS release. In particular, it has been demonstrated that *C. pneumoniae* stimulated NOX and cytochrome c oxidase, whose expression is dependent on the binding of this microorganism to macrophage CD14 receptors and Ca^2+^ influx signaling [40]. Despite ROS production, *C. pneumoniae*, unlike the majority of pathogens, has been demonstrated to survive in macrophages by maintaining a relatively high antioxidant:oxidant ratio, thereby abrogating the bacteria-killing effect of ROS. The ability of *C. pneumoniae* to limit ROS production in macrophages is also supported by the evidence that this microorganism up-regulates the activities of several antioxidant enzyme systems, such as SOD, GPx and γ-glutamylcysteine synthase (γ-GCS) [41].

In endothelial cells, the increased production of superoxide anion may be a consequence of increased expression of COX-2, NOX-2 and NOX-4 paralleled by down-regulation of SOD-1 and thioredoxin-1 following the exposure to *C. pneumoniae* [42]. A similar mechanism has also been observed after the exposure to chlamydial heat shock protein-60 (cHSP60), a known virulence factor of *C. pneumoniae*. cHSP60 has been shown to contribute to endothelial dysfunction by decreasing eNOS expression and NO production through several regulatory mechanisms, including oxidative stress [43,44]. Reduced NO synthesis has also been implicated in cHSP60-induced dysfunction of endothelial progenitor cells, contributing to atherosclerotic lesion progression by impairing vascular repair processes [45]. Lastly, cHSP60 may play a critical role in ox-LDL uptake of endothelial cells, which results from elevated expression of lectin-like oxidized low density lipoprotein receptor 1 (LOX-1) mediated by different molecular pathways, such as NOX activation and eNOS activation phosphatidylinositol-4,5-bisphosphate 3-kinase (PI3K)-protein kinase B (Akt) dependent [46].

In VSMCs, *C. pneumoniae* elicits ROS production in a manner that is independent from NOX activity. Moreover, ROS, largely secreted in the extracellular compartment, affect other vascular cells, inactivating the vasoprotective molecule NO and, thus, contributing to the onset of endothelial dysfunction [47].

At last, in platelets, *C. pneumoniae* stimulates ROS generation and secretion through COX activation and LOX and NOS activation, mediated by protein kinase C (PKC). It has been hypothesized that *C. pneumoniae*, via the lipopolysaccharide, may interact with platelet surface structures and, thus, trigger PKC activity, which, in turn, phosphorylates proteins involved in shape change, aggregation, and ROS production [39].

## 3. *C. Pneumoniae*-Induced Oxidative Stress as a Target for CVD Prevention

At the end of 1990s, the wide number of studies, showing the involvement of *C. pneumoniae* in the pathogenesis of atherosclerosis and CVDs, has opened up new opportunities in the management of adverse cardiovascular outcomes. As a result, numerous clinical antibiotic trials for the prevention of cardiovascular events have been undertaken, but most of them have failed to demonstrate any benefit of anti-chlamydial treatment [3,48]. The failure of clinical trials has been attributed to several factors, as the refractoriness of chlamydial chronic infection to antibiotics, the enrolment of subjects with advanced cardiovascular disease, *etc.* [3,49]. Actually, the lack of beneficial effects on cardiovascular events in clinical antibiotic trials may also be explained by current knowledge on *C. pneumoniae* induced oxidative stress in vascular wall.

Therefore, an approach targeting *C. pneumoniae*-induced oxidative stress by preventing ROS generation may be an attractive strategy for CVD prevention.

To date, several antioxidant compounds have been proposed to prevent the ROS-mediated pro-atherogenic effects, including, as previously reported, LDL oxidation, endothelial dysfunction, VSMC proliferation and migration, and platelet activation induced by *C. pneumoniae* infection.

For example, vitamin E has been shown to prevent the inflammatory state by inhibiting ROS production in endothelial cells, following the exposure to *C. pneumoniae* and oxLDL [36].

Based on the same principle, compounds, such as curcumin and resveratrol, have been considered as possible inhibitors of the enzyme systems generating ROS in the vascular wall. In *C. pneumoniae*-infected monocytes, curcumin and resveratrol have been described as preventing the NOX-mediated ROS production by inhibiting protein kinase C; Resveratrol has also been shown to inhibit ROS production by directly decreasing NOX activity [50]. The effectiveness of resveratrol in preventing the atherogenic changes induced by *C. pneumoniae* has been confirmed in our previous study, as evidenced by the relevant decrease in superoxide anion and oxLDL production, and in the number of foam cells [51].

Also, COX-2 inhibitors such as ibuprofen and diclofenac have been found to reduce *C. pneumoniae*-induced ROS production in monocytes so that they might limit the LDL oxidation as well as the foam cell formation [52].

Interestingly, other investigators have focused their attention on lipid lowering drugs such as statins (coenzyme A reductase inhibitors), since they do not only reduce LDL levels but show antioxidant activity as well [53]. Cerivastatin has been demonstrated to inhibit *C. pneumoniae*-induced oxidative stress in macrophages, VSMCs as well as endothelial cells, thus preventing the inflammation and consequently the atherosclerosis development and progression [54,55]. Fluvastatin has been shown to limit oxLDL uptake in *C. pneumoniae*-infected endothelial cells by inhibiting LOX-1 scavenger receptor activity, which is partially regulated by ROS [56], and, hence, preventing the endothelial dysfunction and progression of atherosclerotic lesion.

An alternative strategy for the prevention of oxidative stress may be to enhance the anti-oxidant cellular defenses [57]. Among the substances able to mimic the biochemical activity of SOD, Mn (III) tetrakis (4-benzoic acid) porphyrin chloride (MnTBAP) has been found to stimulate NOS activity in endothelial cells, down-regulated after the exposure to cHSP60, thus improving the endothelial function by reducing ROS levels and increasing NO bioavailability [43]. Similarly, other free radical scavengers, such as sesamol, have been shown to be effective in inhibiting the proliferation of VSMCs induced by cHSP60 [58].

Nevertheless, concerning the general activity of antioxidants in CVD prevention, early studies have reported encouraging effects, whereas large randomized clinical trials have showed detrimental effects. More recently, a meta-analysis has showed that there is no evidence to support the use of vitamins for the prevention of CVDs [59].

Also, it should be noticed that some antioxidants, mainly vitamins such as vitamin E, may have potential pro-oxidant effects above a certain concentration due to the accumulation of α-tocopherol radicals [60].

Overall, further intense investigations are needed to garner a more complete picture of the effectiveness of these compounds in the prevention of CVDs associated to *C. pneumoniae*. Actually, the existence of numerous antioxidants acting on multi-enzymatic targets involved in oxidative stress makes the identification of effective molecules for the prevention of *C. pneumoniae*-associated CVDs more difficult and demanding.

Therefore an anti-virulence strategy, already considered for the management of infections, may be an intriguing perspective for the prevention of CVDs related to *C. pneumoniae*-mediated oxidative stress. The anti-virulence strategy consists in identifying inhibitors able to specifically target the virulence determinants involved in pathogenetic processes. Strategies that target virulence factors have already been developed for other pathogens, such as, for example, compounds blocking the type III secretion system of *Chlamydia trachomatis*, *Escherichia coli*, *etc.*, or interfering with the quorum sensing of *Pseudomanas aeruginosa*, *Staphylococcus aureus*, *etc.* [61,62,63]. This approach may pave the way to new molecules targeting the virulence determinants involved in *C. pneumoniae*-induced oxidative stress, thus inhibiting the development and progression of atherosclerotic process.

Concerning chlamydial virulence factors, *C. pneumoniae* HSP60, a protein produced by reticulate bodies in chronic infection, has been demonstrated to contribute to LDL oxidation and endothelial dysfunction through oxidative stress [42,43,64] as well as to stimulate VSMC proliferation and inflammatory responses through Toll like receptor activation [65,66,67]. cHSP60 may represent, then, a potential target for prevention of CVDs associated to *C. pneumoniae*-induced oxidative stress.

## 4. Conclusions

*C. pneumoniae*-induced oxidative stress seems to play a crucial role in the pathogenesis of CVDs. The ROS overproduction observed in macrophages, endothelial cells, VSMCs and platelets following the exposure to *C. pneumoniae* is thought to contribute to the initiation, progression and rupture of lipid-rich vascular lesion. A future strategy will be to target chlamydial HSP60, involved in oxidative stress, in order to identify promising candidates for the prevention of *C. pneumoniae*-associated CVDs. 
